# Exosomal PD-L1 promotes tumor growth through immune escape in non-small cell lung cancer

**DOI:** 10.1038/s12276-019-0295-2

**Published:** 2019-08-09

**Authors:** Dong Ha Kim, HyeongRyul Kim, Yun Jung Choi, Seon Ye Kim, Jung-Eun Lee, Ki Jung Sung, Young Hoon Sung, Chan-Gi Pack, Min-kyo Jung, Buhm Han, Kunhee Kim, Woo Sung Kim, Soo Jeong Nam, Chang-Min Choi, Miyong Yun, Jae Cheol Lee, Jin Kyung Rho

**Affiliations:** 1 Department of Pulmonology and Critical Care Medicine, Asan Medical Center, University of Ulsan, College of Medicine, Seoul, 05505 South Korea; 2 Asan Institute for Life Sciences, Asan Medical Center, University of Ulsan, College of Medicine, Seoul, 05505 South Korea; 3 Department of Thoracic and Cardiovascular Surgery, Asan Medical Center, University of Ulsan, College of Medicine, Seoul, 05505 South Korea; 4 Department of Convergence Medicine, Asan Medical Center, University of Ulsan, College of Medicine, Seoul, 05505 South Korea; 5 Department of Biomedical Sciences, Asan Medical Center, AMIST, University of Ulsan College of Medicine, Seoul, 05505 South Korea; 6 Department of Pathology, Asan Medical Center, University of Ulsan, College of Medicine, Seoul, 05505 South Korea; 7 Department of Oncology, Asan Medical Center, University of Ulsan, College of Medicine, Seoul, 05505 South Korea; 8 Department of Bioindustry and Bioresource Engineering, College of Life Sciences, Sejong University, Seoul, 05006 South Korea

**Keywords:** Cancer microenvironment, Immunoediting

## Abstract

Programmed cell death protein-1/programmed cell death ligand-1 (PD-1/PD-L1) pathway blockade is a promising new cancer therapy. Although PD-1/PD-L1 treatment has yielded clinical benefits in several types of cancer, further studies are required to clarify predictive biomarkers for drug efficacy and to understand the fundamental mechanism of PD-1/PD-L1 interaction between host and tumor cells. Here, we show that exosomes derived from lung cancer cells express PD-L1 and play a role in immune escape by reducing T-cell activity and promoting tumor growth. The abundance of PD-L1 on exosomes represented the quantity of PD-L1 expression on cell surfaces. Exosomes containing PD-L1 inhibited interferon-gamma (IFN-γ) secretion by Jurkat T cells. IFN-γ secretion was restored by PD-L1 knockout or masking on the exosomes. Both forced expression of PD-L1 on cells without PD-L1 and treatment with exosomes containing PD-L1 enhanced tumor growth in vivo. PD-L1 was present on exosomes isolated from the plasma of patients with non-small cell lung cancer, and its abundance in exosomes was correlated with PD-L1 positivity in tumor tissues. Exosomes can impair immune functions by reducing cytokine production and inducing apoptosis in CD8^+^ T cells. Our findings indicate that tumor-derived exosomes expressing PD-L1 may be an important mediator of tumor immune escape.

## Lung cancer: Immune suppressant protein promotes tumor growth

An immune suppressant protein expressed by non-small cell lung cancer cells (NSCLC) to facilitate tumor growth could be a valuable therapeutic target. NSCLC is often diagnosed at advanced stages, making treatment challenging. Therapies that inhibit an immune suppressant protein called programmed cell death ligand-1 (PD-L1) have shown promise for other cancers, but how PD-L1 interacts with host and tumor cells in NSCLC needs clarification. In experiments on human cell lines and mice, Jae Cheol Lee and Jin Kyung Rho at the University of Ulsan in Seoul, South Korea, and co-workers found that microvesicles (or ‘exosomes’) released by NSCLC cells carry PD-L1, which interacts with tumor-infiltrating immune cells, inhibiting their activity. The amount of PD-L1 in exosomes directly correlates with PD-L1 expression levels on tumor cell surfaces, providing a useful indication of disease activity.

## Introduction

Lung cancer is the leading cause of cancer death worldwide^1^. Despite advances in the understanding of cancer biology and the development of new therapeutic agents, most cases of lung cancer are diagnosed at an advanced stage and have a poor prognosis, particularly non-small cell lung cancer (NSCLC) cases. For the past 10 years, considerable research has focused on the inhibition of specific targets, such as epidermal growth factor receptor (EGFR) mutation and anaplastic lymphoma kinase (ALK) rearrangement^2^. Although those targeted therapies have improved survival rates among patients with NSCLC, the prognosis of NSCLC remains poor, and new therapeutic approaches are required.

NSCLC was originally not considered to be an immunotherapy-responsive tumor type. That outlook has since changed, however, because of the recent improvement in survival rates due to the use of immune checkpoint inhibitors such as anti-programmed cell death protein-1 (anti-PD-1) or anti-programmed cell death protein ligand-1 (anti-PD-L1) monoclonal antibodies^3,4^. In tumor biology, the PD-1/PD-L1 pathway is a mechanism of adaptive immune escape used by tumor cells in response to endogenous antitumor activity. When PD-L1 expressed on tumor cells binds to PD-1 receptors on immune cells, the interaction leads to the inhibition of CD8^+^ cytotoxic T lymphocyte proliferation, survival, and effector function, thereby inducing the apoptosis of tumor-infiltrating T cells^5^. Despite notable and durable responses in patients, basic and clinical studies are still required to determine the exact mechanism of PD-1/PD-L1 immunotherapy, including the roles of many cofactors, and the relationship between the efficacy of PD-1/PD-L1 immunotherapy and the appropriate selection of patients.

Extracellular vesicles (EVs), also known as microparticles, are small, membrane-enclosed sacs thought to be shed from the surface of healthy or damaged cells under conditions such as cell activation, growth, and apoptosis^6,7^. These membrane-bound vesicular structures contain substantial amounts of biologically active proteins, lipids, and nucleic acids acquired from their parental cells, which they can transport to other cells^8^. Accumulating evidence suggests that EVs are commonly found in blood, urine, saliva, tears, and many other body fluids^9–11^. Many studies have suggested possible roles for EVs as indicators in the diagnosis, prognosis, and surveillance of a variety of health conditions. In particular, tumor cells may release more than one type of membrane-bound vesicle, each with unique morphological traits and functions. These membrane-bound vesicles, such as tumor-derived exosomes, can affect many stages of tumor progression, including the development of the tumor microenvironment^12^, angiogenesis^13,14^, the evasion of immune surveillance^15–17^, invasion and metastasis^18,19^, and the acquisition of aggressive phenotypes^20^ and multidrug resistance^21^.

In this study, we investigated PD-L1 in NSCLC cell-derived exosomes to determine whether the presence of PD-L1 on exosomes reflects the status of PD-L1 on cancer cells and plays a functional role in immune escape. Furthermore, we explored whether similar findings could be observed for exosomes isolated from the plasma of patients with NSCLC.

## Materials and methods

### Cells and cultures

Human NSCLC cell lines (A549, H460, H1975, HCC827, H1650, H820, H358, H1573, H2009, Calu-1, H1299, H3122, H2228, HCC366, and H520), a human bronchial epithelial cell line (BEAS-2B), a human lymphoblastoid T-cell line (Jurkat), and the Lewis lung carcinoma cell line (LLC-1) were purchased from the American Type Culture Collection (ATCC, Manassas, VA, USA). LK2 cells were obtained from the Japanese Collection Research Resources Bank (JCRB, Osaka, Japan). The PC-9 cell line was a kind gift from Dr. Kazuto Nishio (National Cancer Center Hospital, Tokyo, Japan). All resistant cell lines were established in previous studies^22–25^. The PC-9/GR, PC-9/ER, PC-9/WR, HCC827/GR, HCC827/ER, HCC827/CLR, and H1975/WR cell lines have acquired resistance to EGFR-targeted inhibitors, such as gefitinib, erlotinib, WZ4002, and CL-387785; while the HCC827/CR-1 and PC-9/CR-1 cell lines have acquired resistance to cisplatin. The PC-9/GR/WR and PC-9/ER/WR cell lines possess double resistance to gefitinib or erlotinib, respectively, and WZ4002. The H2228/CR cell line has acquired resistance to the ALK inhibitor crizotinib. Tests of the cells for mycoplasma contamination were negative.

### Constructs for the suppression of human PD-L1 (hPD-L1) and overexpression of murine PD-L1 (mPD-L1)

For the generation of lentiviral-based human PD-L1 via CRISPR/Cas9, a single-guide RNA (sgRNA) specifically recognizing and potentially destroying the open reading frame of the hPD-L1 gene was selected using a web-based tool, Benchling (https://benchling.com/). To construct an expression vector for *Streptococcus pyogenes* Cas9 (SpCas9) and an sgRNA specific for the hPD-L1 gene, a lentiviral vector (lentiCRISPR v2, Addgene #52961) was obtained from Addgene (Cambridge, MA, USA), and annealed oligomers (5′-CACCGTCTTTATATTCATGACCTAC-3′ and 5′-AAACGTAGGTCATGAATATAAAGAC-3′) was subcloned using the BsmB1 sites, as previously described^26^. For Sanger sequencing (Macrogen, Inc., Seoul, Korea), the following primers were used for polymerase chain reaction (PCR) analyses: 5′-CAGTTAGAACCACCAAGTCCCA-3′ and 5′-AGGATCTTGGCCTTGTTGAAA-3′ (464 bp for the wild-type PD-L1 gene). The PCR products were cloned using a T-Blunt PCR Cloning kit (SolGent Co., Ltd., Daejeon, Korea). To induce mPD-L1 expression, the pGIPZ-shmPD-L1/Flag-mPD-L1 (mPD-L1) dual expression construct was used to knock down endogenous mPD-L1 expression and reconstitute Flag-mPD-L1 expression, as described elsewhere^27^.

### Generation of stable cells using lentiviral infection

To generate lentivirus-expressing cells, HEK 293T cells were grown to 60–70% confluence prior to transfection. The PD-L1 CRISPR/Cas9 or pGIPZ-shmPD-L1/Flag-mPD-L1 plasmids were transfected using Lipofectamine 2000 (Invitrogen, Carlsbad, CA, USA) according to the manufacturer’s protocol. Six hours after transfection, the medium was changed and subsequently collected at 48 h intervals. The collected medium containing lentivirus was centrifuged to eliminate cell debris and filtered through 0.45 μm filters. Cells were seeded at 50–60% confluence 12 h before infection, and the medium was replaced with medium containing lentivirus and 1 μg mL^–1^ polybrene. After infection for 48 h, the medium was replaced with fresh medium, and infected cells were selected with 2 μg mL^–1^ puromycin (InvivoGen, San Diego, CA, USA). We established two PD-L1 knockout (KO) clones by using H460 cells and LLC-1/mPD-L1 cells expressing Flag-mPD-L1.

### Exosome isolation

Cells (A549, H460, H1975, H460/PD-L1^KO^, and LLC-1/PD-L1) grown to 70–80% confluence were washed twice with phosphate-buffered saline (PBS) and then grown in serum-free RPMI-1640 medium. After 48 h of incubation, the conditioned medium was collected and centrifuged at 300 × *g* for 10 min, 2000 × *g* for 10 min, and 10,000 × *g* for 30 min at 4 °C to thoroughly remove cellular debris. The supernatants were recentrifuged at 100,000 × *g* for 70 min at 4 °C. The pellets were washed with PBS, ultracentrifuged, and resuspended in PBS^28^. Thawed plasma samples were centrifuged using the same method. Isolated exosomes were quantified using a protein assay (Bio-Rad Laboratories Inc., Hercules, CA, USA) and stored at –80 °C until needed.

### Plasma and peripheral blood mononuclear cell (PBMC) isolation

Peripheral blood specimens were collected from 24 patients with lung cancer before surgery (Table S1). Each donor provided informed consent prior to specimen collection. The study was approved by the Institutional Review Board of Seoul Asan Medical Center (2017-0595) and was conducted in accordance with the International Ethical Guidelines for Biomedical Research Involving Human Subjects (CIOMS). The blood samples were delivered to the laboratory and immediately centrifuged at 1000 × *g* for 10 min to separate the plasma from the blood components. The plasma was stored in 2–4 mL aliquots at –80 °C. Peripheral blood obtained from patients with lung cancer and healthy volunteers was used for PBMC isolation on lymphocyte separation medium (Corning, Cambridge, MA, USA). The collected mononuclear cells were resuspended in sorting buffer (PBS supplemented with 1% inactivated fetal bovine serum [FBS]; Gibco BRL, Rockville, MD, USA) and stained with anti-hCD8 antibody (PE-Cy5, HIT8a; BD Biosciences, San Jose, CA, USA). CD8^+^ T cells were selected from the isolated PBMCs by flow cytometry (Becton Dickinson, Franklin Lakes, NJ, USA). PBMCs and CD8^+^ T cells were cultured in RPMI-1640 medium supplemented with 10% inactivated FBS, 100 U mL^–1^ penicillin, and 100 mg mL^–1^ streptomycin at 37 °C and 5% CO_2_.

### Negative-staining electron microscopy (EM) and immuno-EM

For negative-staining EM, purified exosomes were fixed in 2% (v/v) paraformaldehyde for 5 min at room temperature. After fixation, 10 μg of the exosome suspensions was applied to formvar/carbon-coated grids (200 mesh) for 3 min and stained with 2% uranyl acetate. After excess uranyl acetate was removed with filter paper, the grids were examined under a transmission electron microscope (Hitachi H7600, Hitachi, Tokyo, Japan) at 80 kV. For immuno-EM, we used a modified whole-mount immuno-EM method^29^. Briefly, purified exosomes were incubated with anti-PD-L1 (or PD-L1 isotype) antibody in blocking buffer (PBS containing 1% bovine serum albumin [BSA]) for 2 h. Then, 5 μg of the exosome suspensions was applied to formvar/carbon-coated nickel grids (200 mesh) for 3 min. We then washed the grids with five separate drops (50 µL, 10 min per drop) of PBS containing 0.1% BSA, transferred a drop of secondary antibody to the grids for 1 h (anti-mouse immunoglobulin G (IgG) conjugated to a 9–11 nm gold particle (1:100 in PBS containing 0.1% BSA), and repeated the washing procedure. Next, the grids were washed with two separate drops (50 µL) of distilled water. After negative staining with 2% uranyl acetate for 1 min, the specimens were viewed under a transmission electron microscope at 80 kV (Hitachi H-7600, Hitachi).

### Nanoparticle tracking analysis

The size distribution and concentration of isolated exosomes were measured using a NanoSight NS300 instrument (Malvern Instruments, Ltd., Malvern, UK). The data were analyzed using nanoparticle tracking analysis software (NTA version 2.3 build 0017, Malvern Instruments Ltd.). To perform the measurements, samples were diluted 10- to 100-fold in PBS to reduce the number of particles in the field of view to fewer than 100 per frame, and readings were taken in triplicate over 60 s at 10 frames per second at room temperature.

### Immunofluorescence

A 10–20 μg aliquot of exosomes in PBS was incubated with anti-PD-L1 primary antibody (#13684, 1:400, Cell Signaling Technology, Inc., Beverly, MA, USA) and Alexa Fluor 488 goat anti-rabbit IgG (H + L) secondary antibody (Abcam, Cambridge, UK). In addition, exosomes were stained with CellTracker CM-DiI dye (C7000; Thermo Scientific, Rockford, IL, USA), a fluorescent dye that labels the plasma membrane, according to the manufacturer’s instructions. Stained exosomes were visualized under a Zeiss LSM710 confocal microscope (Carl Zeiss Meditec, Jena, Germany).

### Western blot analysis

Cell lysates and exosomes were prepared using EBC lysis buffer (50 mM Tris–HCl [pH 8.0], 120 mM NaCl, 1% Triton X-100, 1 mM EDTA, 1 mM EGTA, 0.3 mM phenylmethylsulfonylfluoride, 0.2 mM sodium orthovanadate, 0.5% NP-40, and 5 U mL^–1^ aprotinin) and centrifuged. Ten micrograms of total cell lysate or exosomes was separated using sodium dodecyl sulfate-polyacrylamide gel electrophoresis and transferred to polyvinylidene fluoride membranes (Invitrogen) for western blot analysis. Membranes were probed using primary antibodies against HSP70 (BD610607, 1:4000) and HSP90 (BD610418, 1:2000; both from BD Biosciences); PD-1 (86163, 1:1000) and PD-L1 (13684, 1:1000; both from Cell Signaling Technology); FLAG (SC807, 1:1000), CTLA4 (SC-376016, 1:1000), and β-actin (SC47778, 1:2000; all from Santa Cruz Biotechnology, Santa Cruz, CA, USA); and CD9 (ab92726, 1:1000, Abcam). Membranes were then incubated with a horseradish peroxidase-conjugated secondary antibody. All membranes were developed using an enhanced chemiluminescence system (Thermo Scientific).

### Measurement of interleukin-2 (IL-2) and interferon-gamma (IFN-γ) production

To quantify IL-2 and IFN-γ production in Jurkat cells, cells were pretreated with each type of exosome for 30 min, followed by incubation in the presence of phorbol 12-myristate 13-acetate (PMA; 50 ng mL^–1^) and ionomycin (500 ng mL^–1^). After 4 h, an enzyme-linked immunosorbent assay (ELISA) was performed with human IFN-γ and human IL-2 (both from BioLegend, CA, USA), according to the manufacturer’s protocols. For determination of intracellular IL-2 and IFN-γ production in CD8^+^ T cells obtained from isolated PBMCs, cells were pretreated with NSCLC cell-derived exosomes or patient-derived exosomes for 30 min, followed by incubation in the presence of PMA (50 ng mL^–1^), ionomycin (500 ng mL^–1^), and brefeldin A (3 μg mL^–1^) for 4 h. After washing with PBS/1% FBS, cells were fixed in 4% (w/v) paraformaldehyde/PBS for 10 min on ice and were then permeabilized using saponin-containing buffer (PermWash, BD Biosciences). To mask PD-1/PD-L1 interaction, we utilized anti-hPD-L1 (B7-H1, 10 μg mL^–1^; BioLegend). Permeabilized cells were stained with anti-human IFN-γ (PE, 25723.11), anti-human IL-2 (FITC, 5344.111), and anti-human CD8 antibodies (PE-Cy5, HIT8a; all from BD Biosciences). Stained cells were then washed twice in permeabilization buffer and once in PBS/1% FBS and were analyzed using a FACScan flow cytometer (Becton Dickinson). FlowJo software (Tree Star, Inc., San Carlos, CA, USA) was used for fluorescence compensation and analysis.

### Flow cytometry analysis

To validate PD-L1 expression in NSCLC cells, resistant cells, and exosomes isolated from the plasma of patients with NSCLC, cells and exosomes were stained with anti-hPD-L1 (PE, 29E.2A3), anti-human CD9 (FITC, HI9a), and isotype control antibodies (all from BioLegend). CD8^+^ T-cell proliferation was measured using a CellTrace™ carboxyfluorescein succinimidyl ester (CFSE) cell division assay kit (Thermo Scientific). CD8^+^ T cells isolated from PBMCs of NSCLC patients were labeled with 2.5 μM CFSE in serum-free medium for 15 min at 37 °C. Staining was quenched with an equal volume of exosome-depleted FBS. CFSE-labeled CD8^+^ T cells (10^4^ cells/well) were pretreated with H460 cell-derived exosomes and activated with 10 μg mL^–1^ anti-CD3 antibody (HIT3a; BD Biosciences), 2 μg mL^–1^ anti-CD28 antibody (CD28.2; Thermo Scientific), and 2 μg mL^–1^ IL-2 (Miltenyi Biotec, Seoul, Korea) for 72 h. To confirm apoptosis of CD8^+^ T cells, CD8^+^ T cells isolated from PBMCs of NSCLC patients were treated with exosomes for 24 h and were then quantified using an annexin V-fluorescein isothiocyanate (FITC)/propidium iodide apoptosis kit (BD Biosciences) in accordance with the manufacturer’s protocols. Briefly, cells were resuspended in annexin V-binding buffer (150 mM NaCl, 18 mM CaCl_2_, 10 nM HEPES, 5 mM KCl, and 1 mM MgCl_2_). FITC-conjugated annexin V (1 μg mL^–1^) and propidium iodide (50 μg mL^–1^) were then added to the cells and incubated for 30 min at room temperature in the dark. All analyses were performed using a FACScan flow cytometer (Becton Dickinson). Data were analyzed with FlowJo software (Tree Star, Inc.).

### Multiplex immunofluorescence

For multiplex immunofluorescence staining using the Opal™ protocol, antigen retrieval was performed in citrate acid buffer by the microwave method for 15 min after boiling, followed by blocking for 10 min in 5% BSA in Tris-buffered saline. Samples were stained with antibodies against CD3 (1:200, PerkinElmer, Waltham, MA, USA) and Ki-67 (1:100, PerkinElmer) using an Opal™ Solid Tumor Immunology kit (OP7TL2001KT, PerkinElmer) according to the standard protocol provided. CD3 and Ki-67 were labeled with Opal 520 and Opal 690 fluorophores. All slides were counterstained with 4′,6-diamidino-2-phenylindole (DAPI) to show nuclei and mounted. Using the Opal™ method, two primary antibodies were sequentially applied to a single slide. Each of the individually stained sections (CD3-opal520, Ki-67-opal690, and DAPI) was utilized to establish the spectral library of fluorophores required for multispectral analysis. Slides were scanned using a Vectra slide scanner (PerkinElmer) under fluorescence conditions.

### Immunohistochemistry

Paraffin-embedded tumor samples were collected from patients and deparaffinized. After rehydration in alcohol, immunohistochemical staining for PD-L1 was performed using anti-PD-L1 antibody (13684, 1:200, Cell Signaling Technology). Immunohistochemical analysis was performed by pathologists at the Asan Medical Center. The expression levels of PD-L1 were scored semiquantitatively according to standard protocols. The percentage of positively stained tumor cells was scored as follows: 0 (<5%), 1 (5–25%), 2 (25–50%), or 3 (>50%). The staining intensity was scored as follows: 0 (no staining), 1 (weak staining), 2 (moderate staining), or 3 (strong staining). Based on the immunohistochemical staining scores, which were obtained by adding the positive percentage scores to the intensity score, tumors were classified as negative (score 0) or positive (score 1–3).

### Mouse experiments

To establish xenograft models, male C57/BL/6 mice (18–20 g, aged 6 weeks) were purchased from Central Lab Animal, Inc. (Seoul, Korea). All experimental procedures followed a protocol approved by the Institutional Animal Care and Use Committee of the Asan Institute for Life Sciences (2017-12-22). Tumors were grown by implanting cells (5 × 10^5^ cells per 0.1 mL) in 50% Matrigel® (BD Biosciences) and subcutaneously injecting them into the right flank of the mice (*n* = 5 mice per group). Exosomes were injected intravenously into the mice three times prior to and after the subcutaneous implantation of the tumor cells. To estimate the tumor sizes, the length (*L*) and width (*W*) of each tumor were measured using calipers, and the tumor volume was calculated as (*L* × *W*^2^)/2.

### Statistics

Data are presented as the means ± standard deviations. *P-*values were determined using unpaired or paired *t*-tests between groups (GraphPad Prism software, GraphPad Software Inc., San Diego, CA, USA).

## Results

### Secretion of PD-L1-containing exosomes from NSCLC cells

**Fig. 1:**
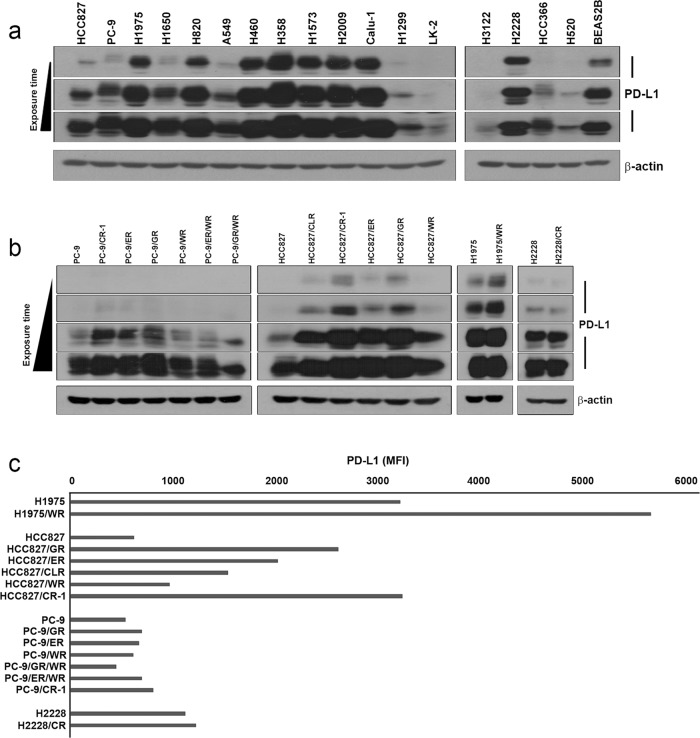
The expression of PD-L1 in NSCLC cell lines and cells with acquired resistance to various anticancer drugs. **a** The basal expression of PD-L1 in NSCLC cells was determined by western blot analysis. **b**, **c** PD-L1 expression in parental and resistant cells was determined by western blot analysis and flow cytometry. MFI, median fluorescence intensity; CR-1, cisplatin resistance; GR, gefitinib resistance; ER, erlotinib resistance; WR, WZ4002 resistance; CLR, CL-387785 resistance; CR, crizotinib resistance

**Fig. 2:**
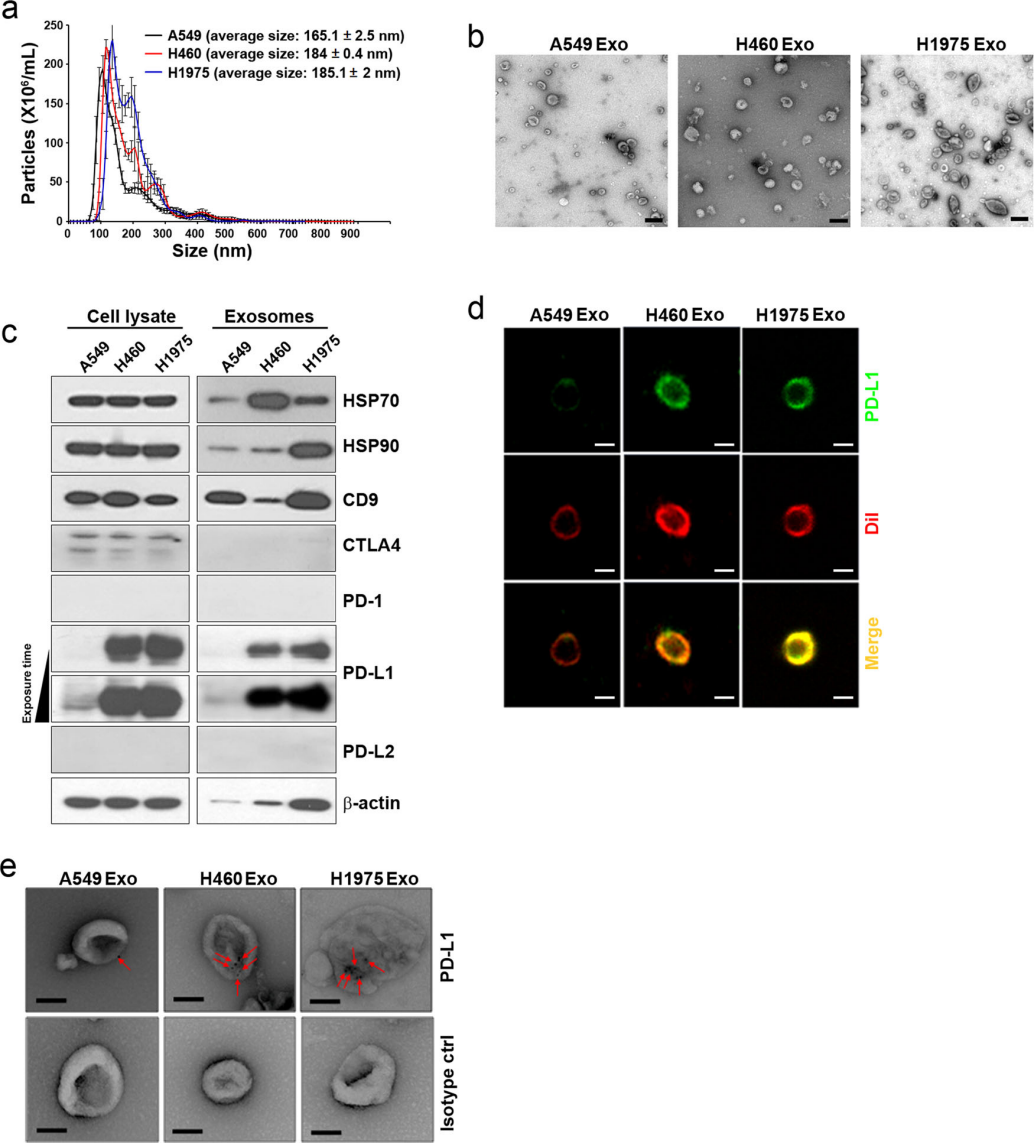
Identification of PD-L1 in exosomes isolated from lung cancer cell lines. Exosomes were isolated by using ultracentrifugation as described in the methods. **a** Nanoparticle tracking analysis (NTA) of exosomes derived from A549, H460, and H1975 cells. **b** Representative electron micrograph of exosomes. Scale bars: 100 nm. **c** Western blot analysis was performed with 10 μg of exosomal protein to validate the expression of PD-L1 and exosome biomarker proteins. **d** Representative immunofluorescence images of exosomes. Exosomes were immunostained with anti-PD-L1 (green fluorescence) and DiI (red fluorescence). Scale bars: 100 nm. **e** Representative immuno-electron micrograph of exosomes. The red arrows indicate PD-L1 expression. Scale bars: 100 nm

Although PD-L1 expression may vary according to different tumor microenvironments and tumor types, most of the NSCLC cells that we tested expressed PD-L1 (Fig. 1a). PD-L1 expression was dramatically increased in cells with acquired resistance to anticancer drugs compared with that in parental cells (Fig. 1b, c), corroborating previous reports that associated PD-L1 expression with advanced tumor stage^30,31^. We selected three cell lines according to their differential expression levels of PD-L1. To identify the presence of PD-L1 within cancer cell-derived exosomes, we isolated exosomes from cell culture media using ultracentrifugation. The number and size distribution of exosomes did not differ among the cell lines, and we confirmed the exosomes by EM (Fig. 2a, b). In addition, we detected known exosome markers, including CD9, Hsp70, and Hsp90. We found PD-L1 in the isolated exosomes, and the amount of PD-L1 in the exosomes was proportional to the quantity of PD-L1 expression on the cells from which the exosomes were derived (Fig. 2c, d). Furthermore, PD-L1 was especially abundant in exosomes (Supplementary Fig. 1). The plasma membrane localization of PD-L1 in the exosomes was confirmed by confocal microscopy (Fig. 2e). Together, these results show that PD-L1 is present in exosomes secreted by cancer cells and that its abundance in these exosomes reflects its expression on the cancer cells.

### PD-L1-containing exosomes inhibit T-cell activity

**Fig. 3:**
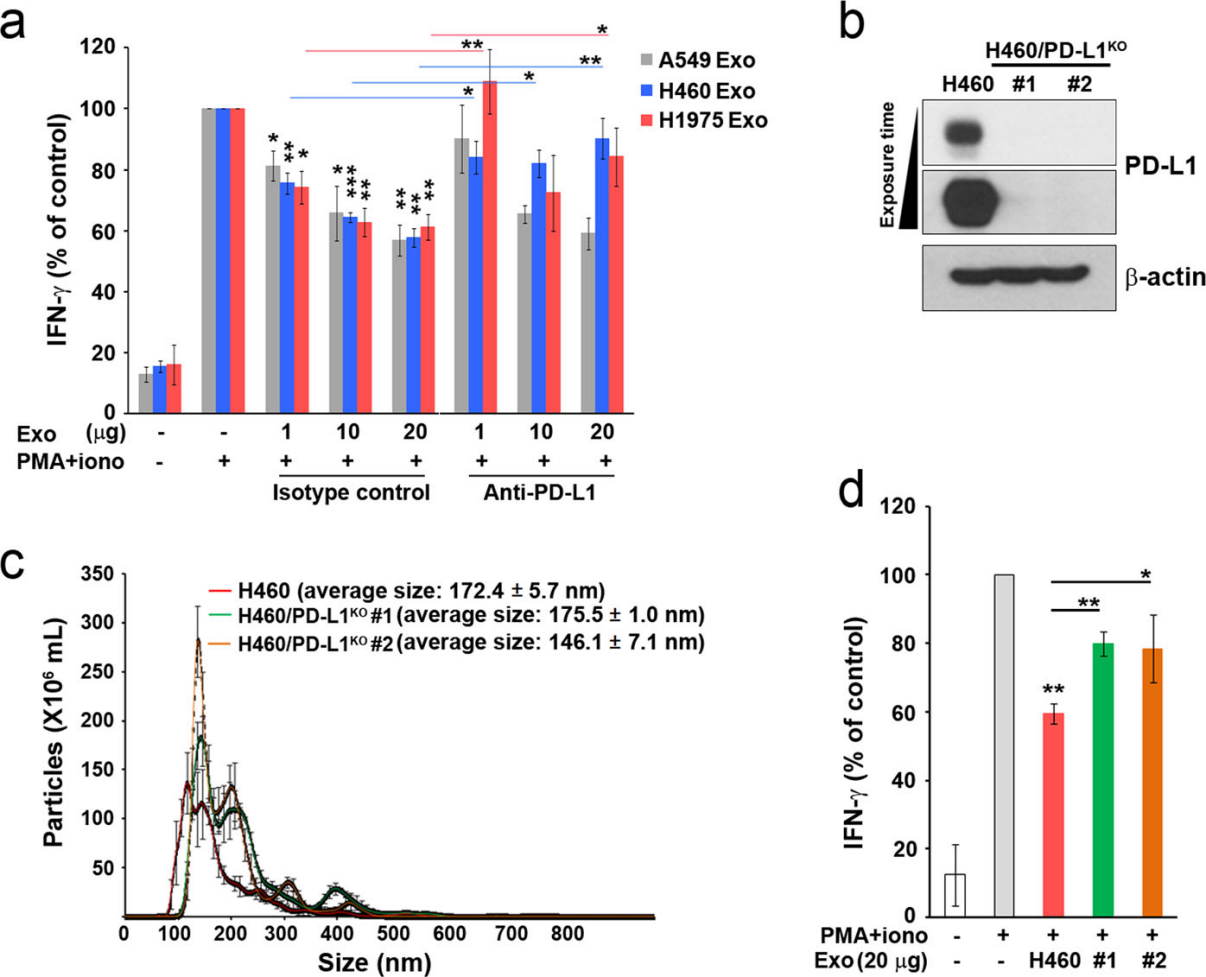
PD-L1-containing exosomes inhibit INF-γ production in Jurkat cells. **a** Jurkat cells were treated with the indicated exosomes for 4 h in the presence of PMA (50 ng mL^–1^) and ionomycin (500 ng mL^–1^) after PD-L1 masking within the exosomes through treatment with control or anti-PD-L1 antibodies for 30 min. IFN-γ production was measured by ELISA. **b** H460/PD-L1^KO^ cells were generated as described in the methods. PD-L1 expression was confirmed by western blot analysis. **c** Nanoparticle tracking analysis (NTA) of H460 cell-derived and H460/PD-L1^KO^ cell-derived exosomes. **d** Jurkat cells were treated with H460-derived or H460/PD-L1^KO^ cell-derived exosomes for 4 h in the presence of PMA (50 ng mL^–1^) and ionomycin (500 ng mL^–1^), and IFN-γ production was then measured by ELISA. The results are from three experiments. All data are presented as the means ± standard deviations. *P < 0.05, **P < 0.005, and ***P < 0.0005 by a paired two-tailed Student’s *t*-test

To determine whether PD-L1-containing exosomes can affect T-cell activity through PD-1/PD-L1 interaction, we examined IL-2 and INF-γ production by T cells following treatment with exosomes. Although treatment with PMA plus ionomycin is well known to induce IL-2 and INF-γ production by T cells, we found that it increased IL-2 and INF-γ production only in Jurkat cells and not in MOLT4 cells or CCRF-CEM cells (Supplementary Fig. 2). PD-1 expression was minimal in untreated Jurkat cells and was increased in those cells after treatment with PMA plus ionomycin (Supplementary Fig. 3). Therefore, we used Jurkat cells to evaluate the response of T cells to treatment with exosomes containing PD-L1. When we treated these cells with PD-L1-containing exosomes, INF-γ production decreased in a dose-dependent manner, although the production of IL-2 did not change in response to the exosomes (Fig. 3a and Supplementary Fig. 4). Next, to elucidate whether those trends were caused by PD-1/PD-L1 interaction, we incubated exosomes with anti-PD-L1 neutralizing antibody. H460 cell-derived and H1975 cell-derived exosomes with high PD-L1 levels significantly reversed the exosome-induced reduction of INF-γ production (Fig. 3a), whereas A549 cell-derived exosomes with low PD-L1 levels did not. To confirm these findings, we used CRISPR/Cas9 gene editing to knockout *PD-L1* in H460 cells (Supplementary Fig. 5). We acquired two PD-L1-deficient clones that secreted exosomes without PD-L1 (Fig. 3b, c). Consistent with our results described above, the capability of exosomes without PD-L1 to decrease the production of INF-γ in Jurkat cells was diminished compared with that of H460 cell-derived exosomes (Fig. 3d), suggesting that PD-L1-containing exosomes may modulate IFN-γ signaling in T cells through PD-1/PD-L1 interaction.

### PD-L1-containing exosomes promote tumor growth

**Fig. 4:**
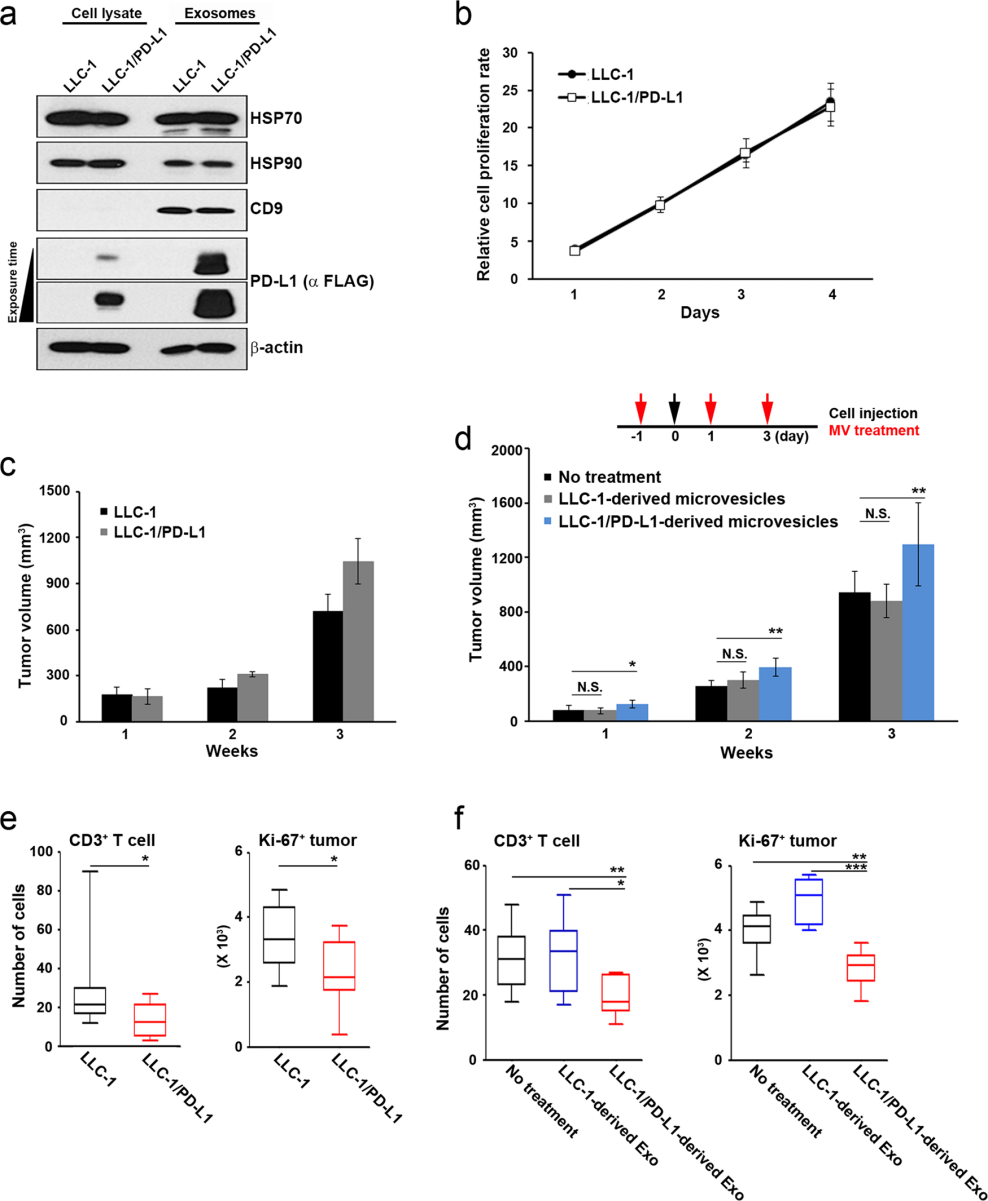
The effect of PD-L1-containing exosomes on tumor growth in vivo. **a** Production of mPD-L1-containing exosomes by LLC-1 cells. Stable LLC-1/PD-L1 cells were generated by infection with pGIPZ-shmPD-L1/Flag-mPD-L1. PD-L1 expression levels in cell lysates (10 μg) and exosomes (10 μg) were determined by western blot analysis. **b** The cell proliferation rate was measured using a cell counter. **c** A xenograft tumor model was established by injecting LLC-1 or LLC-1/PD-L1 cells into the flank region of mice (*n* = 10). **d** Exosomes (50 μg 100 μL^–1^) were injected into the tail vein of mice (*n* = 10) three times before and after the injection of LLC-1 cells. Tumor volumes were measured on the indicated day. **e**, **f** The numbers of tumor-infiltrating T cells (CD3+ cells) and proliferating tumor cells (Ki-67+ cells) were counted by using multiplex immunofluorescence in tumors harvested from the tumor-bearing mice described in (**c**) and (**d**). All data are presented as the means ± standard deviations. *P < 0.05 and **P < 0.005 by a paired two-tailed Student’s *t*-test

To determine the role of PD-L1 in tumor growth in vitro and in vivo, we established stable murine LLC-1 cells with forced expression of mPD-L1. The parental murine LLC-1 cells do not express PD-L1. Both the PD-L1-transduced LLC-1 cells and the exosomes derived from those cells contained PD-L1 (Fig. 4a). Expression of PD-L1 did not affect cell growth in vitro (Fig. 4b), whereas PD-L1-transduced LLC-1 cells grew faster than the parental LLC-1 cells when the cells were subcutaneously injected into mice (Fig. 4c). However, expression of PD-L1 did not affect tumor growth in the immunodeficient mouse model, similar to the results for tumor growth in vitro (Supplementary Fig. 6).

We next investigated whether PD-L1-containing exosomes can affect tumor growth in vivo. To answer this question, we injected PD-L1-containing exosomes intravenously into mice three times prior to and after the subcutaneous implantation of LLC-1 cells. PD-L1-containing exosomes induced a higher growth rate than either parental LLC-1-derived exosomes or control treatment (Fig. 4d). These effects of PD-L1 expression or PD-L1-containing exosomes on tumor growth were accompanied by decreased proliferation (as measured by quantitative analysis of Ki-67 staining in the tumor cells) and a reduction in tumor-infiltrating lymphocytes (as measured by quantitative analysis of CD3 staining in the tumor microenvironment; Supplementary Fig. 7 and Fig. 4e, f). Our observations suggest that PD-L1-containing exosomes can promote tumor growth in vivo.

### Effects of tumor-derived exosomes on T cells in patients with NSCLC

**Fig. 5:**
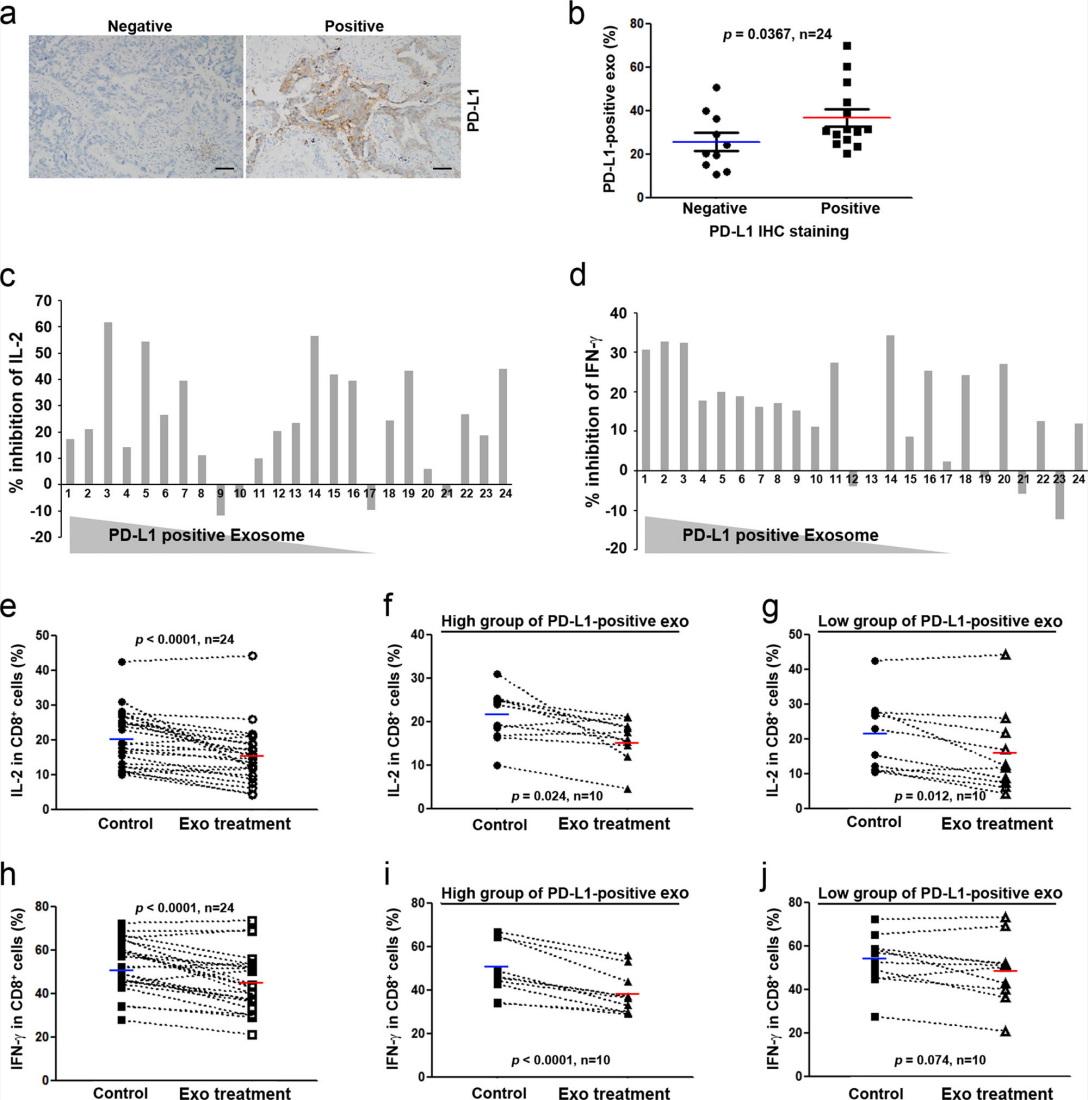
Clinical implication of exosomes derived from the serum of patients with NSCLC. **a** Representative images of immunohistochemistry for PD-L1. Scale bars: 50 μm. **b** Relationship between the number of PD-L1-positive exosomes and PD-L1 expression in tumor tissue. Each dot represents one individual; the horizontal lines represent the medians. **c**–**j** Inhibitory effect of exosomes on IL-2 and IFN-γ production in CD8^+^ T cells isolated from 24 patients with NSCLC. CD8^+^ T cells were stimulated with PMA (50 ng mL^–1^), ionomycin (500 ng mL^–1^), and brefeldin A (3 μg mL^–1^) for 4 h after treatment with autologous exosomes from the same patient. Cells were stained with anti-CD8 antibody, fixed, and permeabilized, followed by intracellular staining with anti-human IL-2 and IFN-γ antibodies and analysis using a FACScan flow cytometer. **c**, **d** Inhibition of IL-2 and IFN-γ production in individual samples according to treatment with exosomes. **e**, **h** Summary of the analysis of IL-2 and IFN-γ production inhibition. Summary of the analysis of IL-2 and IFN-γ inhibition in patients with a high proportion of PD-L1-positive exosomes (**f**, **i**) and patients with a low proportion of PD-L1-positive exosomes (**g**, **j**). *P* = 0.0367 by an unpaired two-tailed Student’s *t*-test (B); *P* < 0.0001, *P* = 0.024, *P* = 0.012, and *P* = 0.074 by a paired two-tailed Student’s *t*-test (**e**–**j**)

To determine the clinical relevance of our findings, we analyzed PD-L1 levels in tissues and in circulating exosomes isolated from patients who underwent surgery for NSCLC. The exosomes from the patients showed a relatively similar size distribution to the exosomes derived from cancer cells in vitro, within 100–200 nm (Supplementary Fig. 8). However, although the proportion of PD-L1-positive exosomes from each patient varied between 10 and 70% (Supplementary Fig. 9), this proportion was correlated with the PD-L1 expression level in the cancer tissues from the same patients (Fig. 5a, b). Next, we examined cytokine production from CD8^+^ T cells following treatment with autologous exosomes. For those experiments, we used CD8^+^ T cells sorted from PBMCs to prevent dilution of the exosomes. Autologous exosome treatment inhibited IL-2 and IFN-γ production in CD8^+^ T cells of most patients (Fig. 5c–e, h). To further evaluate whether the proportion of PD-L1-positive exosomes was associated with the reduction in IL-2 and IFN-γ production, we divided the patients’ samples according to the proportion of PD-L1-positive exosomes (low, *n* = 10, <26% PD-L1-positive exosomes; high, *n* = 10, >31% PD-L1-positive exosomes) and analyzed the inhibition of cytokine production. The samples treated with a high proportion of PD-L1-positive exosomes displayed significantly reduced IL-2 and IFN-γ production (Fig. 5f, i), whereas those treated with a low proportion of PD-L1-positive exosomes exhibited significantly reduced IL-2 production but only a small, nonsignificant reduction in IFN-γ production (Fig. 5g, j).

**Fig. 6:**
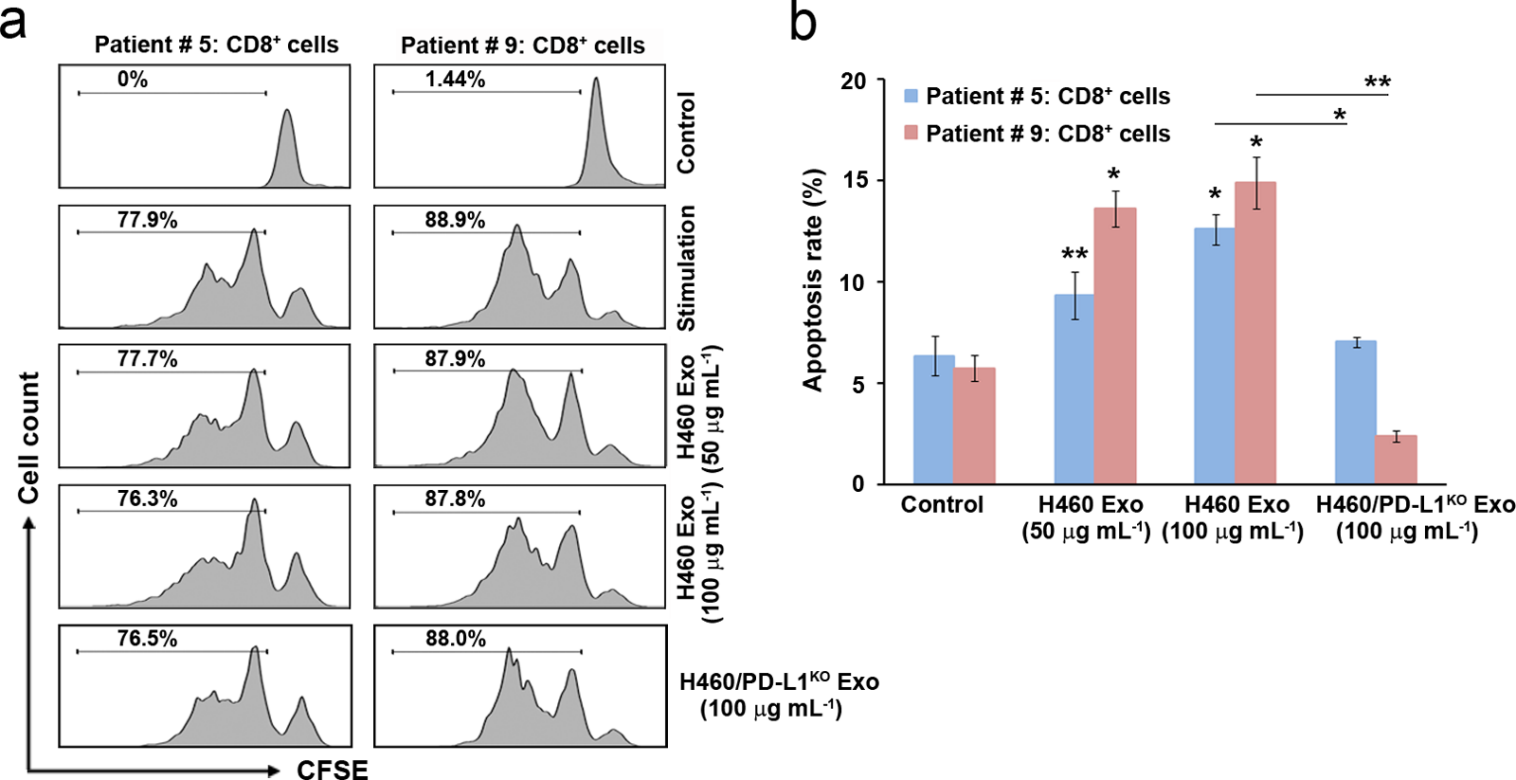
Induction of apoptosis by PD-L1-positive exosomes. **a** CD8^+^ T cells (from patients #5 and #9) were labeled with 2.5 μM CFSE and cultured with H460 cell-derived exosomes or H460/PD-L1^KO^ cell-derived exosomes in the presence of 10 μg mL^–1^ anti-CD3, 2 μg mL^–1^ anti-CD28, and 2 μg mL^–1^ anti-IL-2 for 72 h. **b** CD8^+^ T cells were incubated with H460 cell-derived exosomes or H460/PD-L1^KO^ cell-derived exosomes for 24 h and then double stained with Annexin V-FITC (1 μg mL^–1^) and propidium iodide (50 μg mL^–1^). Analyses were performed using a FACScan flow cytometer. All data are presented as the means ± standard deviations. *P < 0.05 and **P < 0.005 by a paired two-tailed Student’s *t*-test

Earlier works demonstrated that PD-L1 can deliver an inhibitory signal to PD-1-expressing T cells, leading to T-cell dysfunction by inducing T-cell apoptosis, anergy, and exhaustion^32,33^. We examined the proliferation and apoptosis of CD8^+^ T cells following treatment with PD-L1-positive exosomes. CD8^+^ T cells proliferated in response to CD3/CD28 stimulation, but treatment with exosomes did not affect their proliferation (Fig. 6a). Exosome treatment did, however, decrease the total number of CD8^+^ T cells in a dose-dependent manner (Supplementary Fig. 10). Consistent with these results, treatment with exosomes significantly induced the apoptosis of CD8^+^ T cells, an effect that was reduced when the cells were treated with PD-L1-negative exosomes (Fig. 6b), indicating that PD-L1-positive exosomes promote the apoptosis of CD8^+^ T cells through PD-1/PD-L1 interaction.

## Discussion

Blockade of the PD-1/PD-L1 pathway can reverse the tumor microenvironment and induce an endogenous antitumor immune response. Indeed, PD-1/PD-L1 immunotherapy has shown benefits for patients with NSCLC in clinical trials^34,35^. Although the exact mechanisms of tumor immune escape through PD-1/PD-L1 interaction are still unclear, tumors with a higher mutational burden, such as melanomas and lung cancers, seem to be more responsive to PD-1/PD-L1 immune checkpoint inhibitors^36,37^. Given the high incidence and mortality of lung cancer, the success of these inhibitors in lung cancer treatment has greatly impacted cancer therapy.

Recently, Ludwig et al. (2017) showed that exosomes in the plasma of patients with head and neck cancer carry immunosuppressive molecules and interfere with immune cell functions. Furthermore, the same group of researchers revealed that the PD-L1 levels on exosomes are associated with disease progression in patients with head and neck cancer, suggesting that circulating PD-L1 exosomes are a useful marker of disease activity^38^. Accordingly, we found that cancer cell-derived exosomes express PD-L1 and that those exosomes can support tumor progression in lung cancer. In addition, PD-L1 was present on exosomes isolated from the blood of patients with NSCLC, and PD-L1-positive exosomes played an important role in tumor immune evasion through the inhibition of cytokines and the induction of apoptosis in CD8^+^ T cells. Considering that cancer immunotherapy is the most actively evolving therapy for lung cancer, we believe that this study has some important findings, which should be further pursued by confirmatory and extended studies.

EVs, including microvesicles (MVs) and exosomes, can participate in intercellular communication by transferring their bioactive molecules, including proteins, RNA, DNA, and lipids, between cells^39^. Emerging evidence has demonstrated that oncoprotein-containing MVs or exosomes can contribute to malignant phenotypes, drug resistance, and metastasis^20,40,41^. Oncoproteins, including EGFR, EGFRvIII, and the receptor tyrosine kinase MET, can be delivered by MVs or exosomes into recipient cells, where they can induce signaling pathways to promote aggressive phenotypes. Although most components within these vesicles perform their function after they are delivered into recipient cells, we found that the interaction between PD-1 on CD8^+^ T cells and PD-L1 on tumor-derived exosomes promotes tumor immune escape by impairing the function and inducing the apoptosis of CD8^+^ T cells. It has already been shown that exosomes secreted from Epstein-Barr virus-immortalized human B lymphoblastoid cells contain FasL and play a role as inducers of immune tolerance^42^. Thus, membrane proteins on the surface of exosomes are capable of performing their function through direct protein–protein interactions.

PD-L1 expression is elevated in several types of cancer, including melanoma, glioblastoma, lung cancer, renal cancer, gastric cancer, colorectal cancer, pancreatic cancer, breast cancer, and cervical and ovarian cancers^43^. The induction of PD-L1 expression in cancers is associated with oncogenic pathways, including the PTEN-PI3K-Akt and STAT3 pathways^44^. In addition, expression of PD-L1 is often correlated with resistance to anticancer therapies, including conventional and targeted drugs^45–47^. Similarly, we also found that PD-L1 expression was induced in cells with acquired resistance to various anticancer drugs. Although the mechanisms of induced PD-L1 expression in cells with acquired resistance to anticancer drugs are not clear from the present study, the induction of PD-L1 expression may affect cancer growth, drug resistance, and immune escape through PD-L1 signaling as well as through exosomes derived from cancer cells. Further studies are needed to identify the mechanisms involved in induced PD-L1 expression and the consequent effects on cancer progression.

The mechanisms of immune suppression by PD-1/PD-L1 interaction in tumors are far more complicated. To date, accumulating evidence suggests that tumor-associated PD-L1 can induce the apoptosis of activated T cells^32^, facilitate T-cell anergy and exhaustion^5,48^, enhance the function of regulatory T cells^49,50^, inhibit the proliferation of T cells^51,52^, and restrain impaired T-cell activation and IL-2 production^53,54^. Similarly, our results showed that exosomes containing PD-L1-induced apoptosis and inhibited cytokine production in CD8^+^ T cells, implying that exosomes containing PD-L1 may play a role similar to that of tumor cell-associated PD-L1. We believe that exosomes containing PD-L1 can be more potent than tumor cell-associated PD-L1 in facilitating escape from antitumor immunity because exosomes can be widespread and may attach to their target cells more easily than tumor cells. To better understand the role of PD-L1-containing exosomes in tumor immune escape, the function of PD-L1-containing exosomes must be validated in various immune cells, such as dendritic cells, B cells, regulatory and effector T cells, NK cells, and macrophages. We are currently pursuing such validation efforts.

Anti-PD-1/PD-L1 therapy has generated significant clinical benefits in patients with NSCLC^3,4^. However, the use of PD-L1 expression as a predictive biomarker for anti-PD-1/PD-L1 therapy is controversial because of the challenging nature of the assay and uncertainty about its clinical efficacy. In addition, PD-L1 expression may differ according to the time and site of biopsy and the previous therapies received by the patient. Temporal and spatial tumor heterogeneity could result in inappropriate decisions about the use of immune checkpoint inhibitors. The difficulty of performing repeated biopsies is also an important problem. Considering those problems, liquid biopsy can offer several potential advantages because it is noninvasive and convenient, can be performed at multiple time points, and can overcome the problems associated with tumor heterogeneity. Using our data, we confirmed the existence of PD-L1-positive exosomes in the serum of patients with NSCLC, as well as a positive correlation between the number of PD-L1-positive exosomes and the level of PD-L1 expression in the tumor tissue, although tumor-specific exosomes could not be distinguished in the present study. However, Chen et al. (2018) recently proposed that exosomal PD-L1 is associated with an anti-PD-1 response in melanoma, and we are also performing further research to evaluate whether PD-L1-positive exosomes in plasma can be used as a surrogate marker of PD-L1 expression in tumor tissue to predict the efficacy of PD-1/PD-L1 immunotherapy. In summary, tumor cells release PD-L1 in exosomes, and these exosomes promote tumor growth, inhibit CD8^+^ T-cell activity, and induce the apoptosis of CD8^+^ T cells. To improve the efficacy of PD-1/PD-L1 immunotherapy, a better understanding of the pathophysiological functions of exosomes in immune evasion and further study of the clinical implications of those functions is required.

## Supplementary information

Supplementary Information

## Data Availability

All data generated or analyzed during this study are included in this published article and the supplementary information.
