# Supplementary material for: Exosomal PD-L1 promotes tumor growth through immune escape in non-small cell lung cancer
Source: Exp Mol Med. 2019 Aug 9;51(8):94. doi: 10.1038/s12276-019-0295-2 (PMC6802663; doi:10.1038/s12276-019-0295-2)
Supplement: Supplementary file 1 — Supplementary Information [file 12276_2019_295_MOESM1_ESM.pdf]

Supplemental Information

Exosomal PD-L1 promotes tumor growth through immune escape in non-small cell lung cancer

Dong Ha Kim, Hyeong Ryul Kim, Yun Jung Choi, Seon Ye Kim, Jung-Eun Lee, Ki Jung Sung, Young Hoon Sung, Chan-Gi Pack, Min-kyo Jung, Buhm Han, Kunhee Kim, Woo Sung Kim, Soo Jeong Nam, Chang-Min Choi, Miyong Yun, Jae Cheol Lee, Jin Kyung Rho

Supplemental Materials and Methods

Cells and cultures

CCRF-CEM cell line, MOLT-4 cell line, and Lewis lung carcinoma cell line (LLC-1) were purchased from the American Type Culture Collection (ATCC, Manassas, VA, USA). The cells were cultured in 10% fetal bovine serum (FBS), 100 U mL^{-1} penicillin, and 100 mg mL^{-1} streptomycin (Invitrogen, Carlsbad, CA, USA) at 37 °C in an atmosphere with 5% CO_2. Tests of the cells for mycoplasma contamination were negative.

Cell proliferation assay

To validate the proliferation according to PD-L1 expression, cells were seeded into 96-well plates overnight and then treated with the indicated exosomes. The viable cell counts were determined using an ADAM-MC automatic cell counter (NanoEnTek, Seoul, Korea).

Supplementary Table1. Clinical information about the patients with NSCLC

**ID**	**Age (years)**	**Sex**	**Tumor type**	**PD-L1 positive exosome (%)**	**PD-L1 in IHC**
1	43	M	ACC	69.8	+
2	64	M	ACC	60.4	+
3	59	M	SqCC	52.9	+
4	72	M	ACC	50.6	_
5	61	F	ACC	43.8	+
6	62	F	ACC	39.8	_
7	73	M	ACC	38.8	+
8	70	M	SqCC	36.1	_
9	64	M	SqCC	31.5	+
10	72	M	ACC	31.3	+
11	61	M	ACC	30.6	+
12	53	F	ACC	30.1	+
13	74	M	SqCC	29.1	+
14	57	M	ACC	28.9	_
15	63	M	SqCC	26.5	+
16	62	M	ACC	24.5	+
17	72	M	SqCC	24.1	_
18	50	F	ACC	23.5	+
19	54	M	ACC	20.4	+
20	65	M	SCLC	20.2	_
21	70	F	ACC	19.4	_
22	75	M	ACC	14.9	_
23	66	F	ACC	11.8	_
24	66	M	ACC	10.6	_

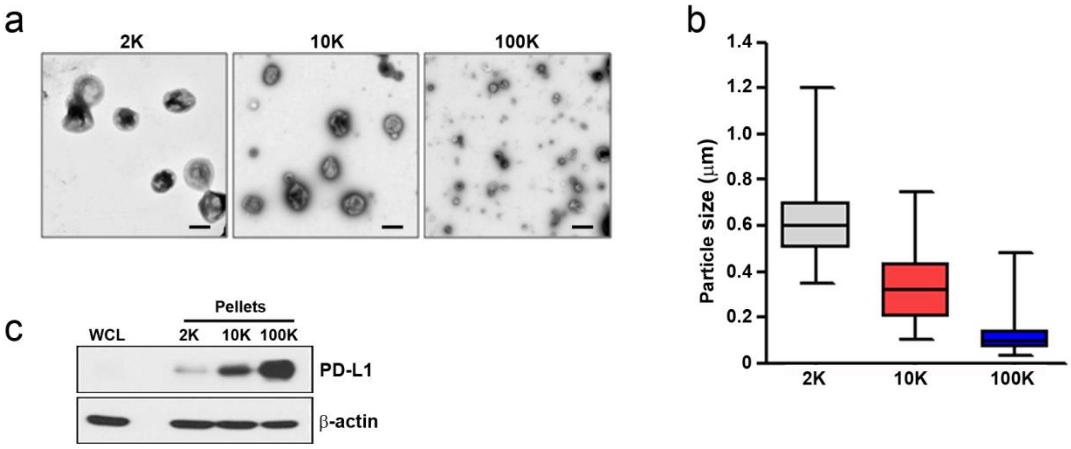

Supplementary Figure 1. Determination of PD-L1 expression according to the size distribution of extracellular vesicles (EVs). H460 cells at 80% confluence were washed twice with PBS and then grown in serum-free media. After 48 h, the conditioned medium was collected, and EVs were pelleted using multiple centrifugation steps (2K = 2,000 *g*; 10K = 10,000 *g*; 100K = 100,000 *g*). **a** Representative electron micrograph of the EVs. **b** Analysis of the size distribution of the EVs. **c** PD-L1 expression was determined by Western blot analysis. All data represent the mean ± standard deviation. Scale bars: 500 nm.

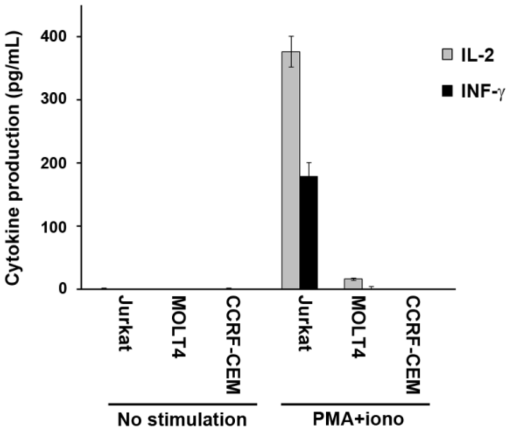

Supplementary Figure 2. IL-2 and INF-γ production in T cells. All cells were stimulated with PMA/ionomycin for 4 h. Cytokines were measured using ELISA.

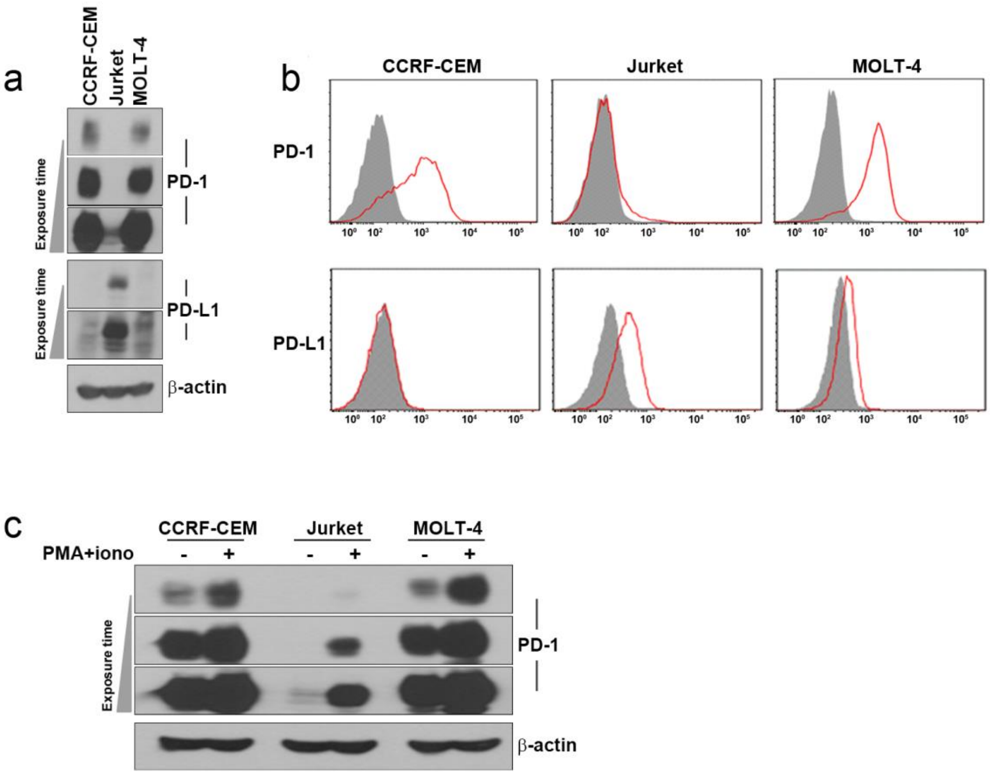

Supplementary Figure 3. PD-1/PD-L1 expression in T cells. **a** The basal expression of PD-1/PD-L1 was determined by Western blot analysis. **b** Cells were stained with the PE-anti-human PD-1 and PE-anti-human PD-L1 (open histograms, red line) and analyzed using a FACScan flow cytometer. Negative controls were stained with PE-mouse IgG (filled histogram, gray). **c** Cells were stimulated with PMA/ionomycin for 4 h, and then PD-1 expression was determined by Western blot analysis.

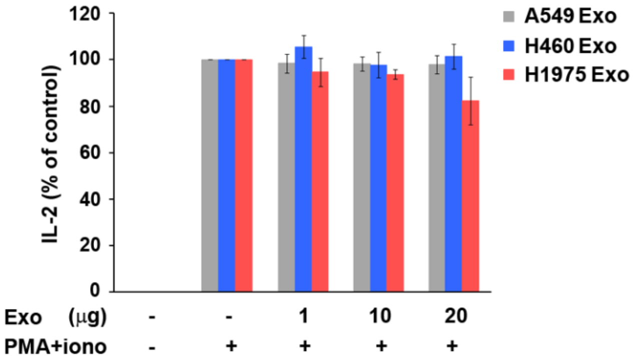

Supplementary Figure 4. IL-2 production in Jurkat cells. Cells were stimulated with PMA/ionomycin in the presence of the indicated exosomes for 4 h. IL-2 production was measured by ELISA analysis.

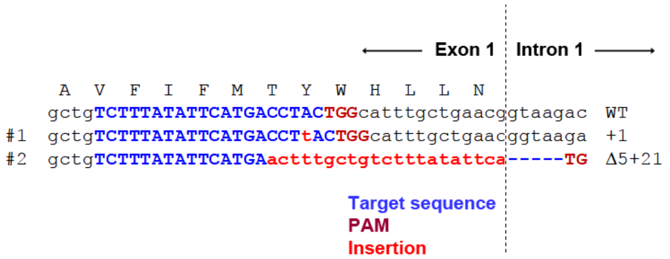

Supplementary Figure 5. Generation of constructions for PD-L1 knockout. Constructions for PD-L1 knockout were generated as described in the supplemental materials and methods. In this study, two clones were selected by sequencing.

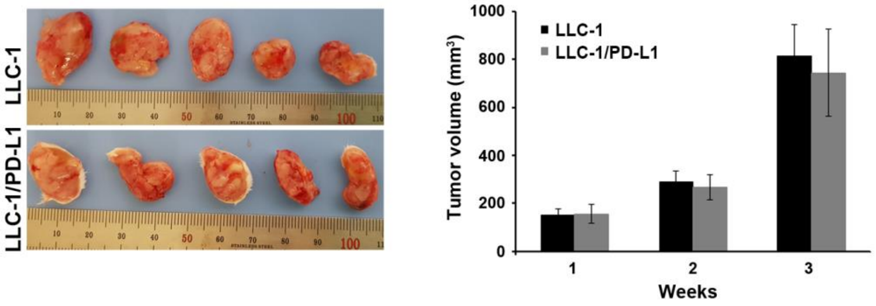

Supplementary Figure 6. The effects of PD-L1 expression on tumor growth in immunodeficient mice. A xenograft tumor model was established by injecting LLC-1 or LLC-1/PD-L1 cells into the flank region of mice (*n* = 5). Tumor volume was measured on the indicated day. All data represent the mean ± standard deviation.

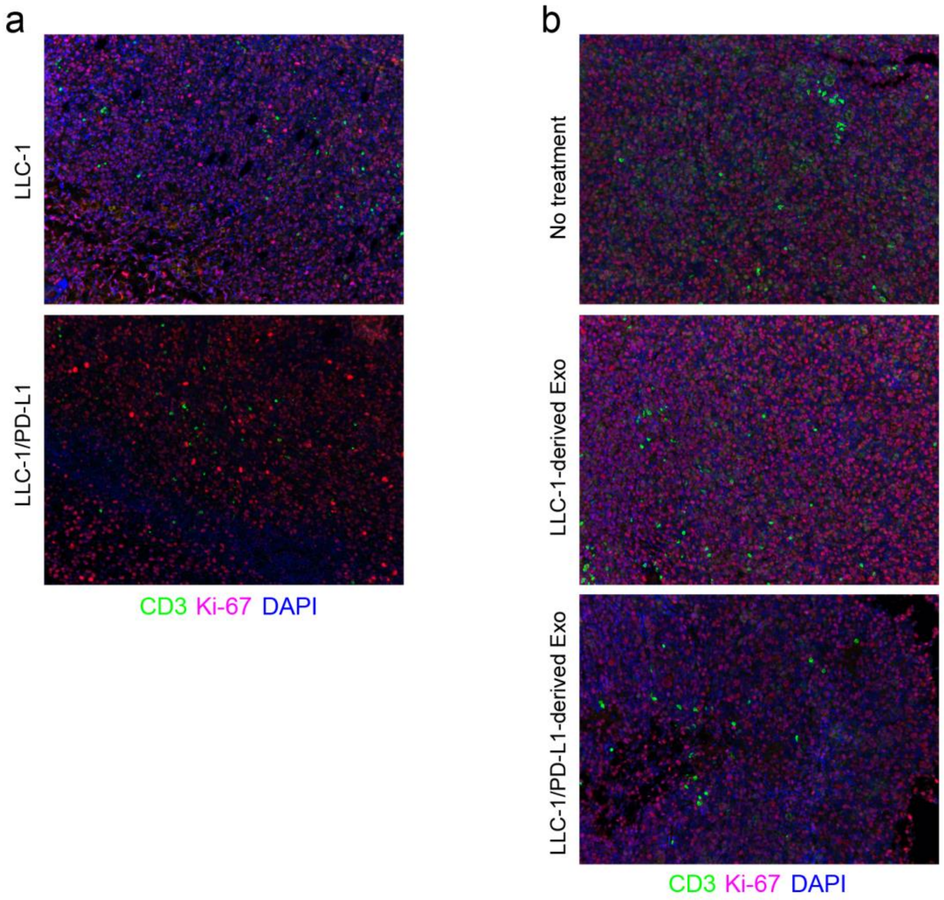

Supplementary Figure 7. Representative immunofluorescence images of Figure 4e and f. Xenograft tissues were stained with CD3, Ki-67, and DAPI, as described in the materials and methods.

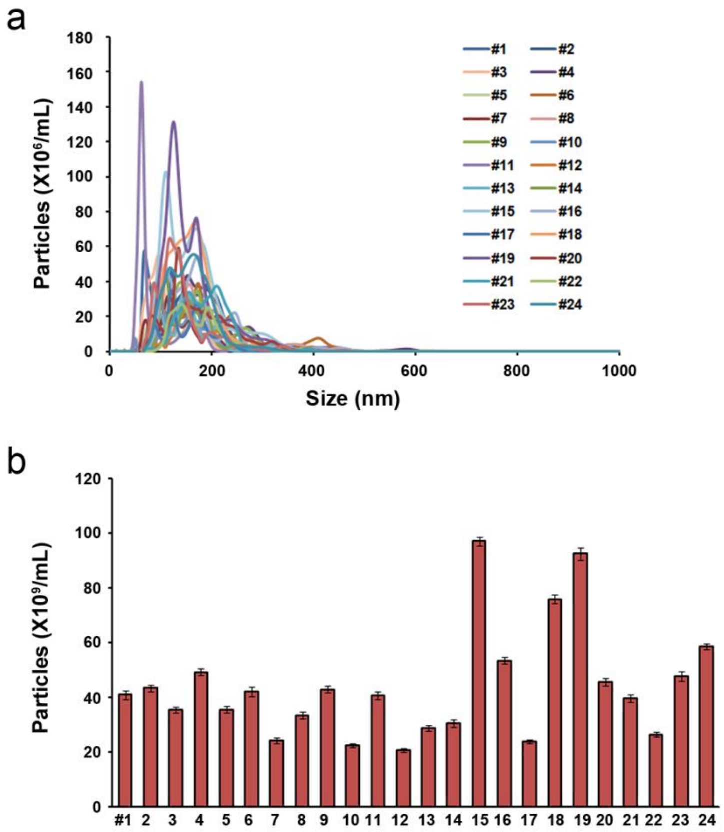

Supplementary Figure 8. Analysis of isolated exosomes from 24 patients with NSCLC.

Exosomes were isolated from 24 patients with NSCLC. The size distribution (**a**) and amount (**b**) of the exosomes were determined by nanoparticle tracking analysis.

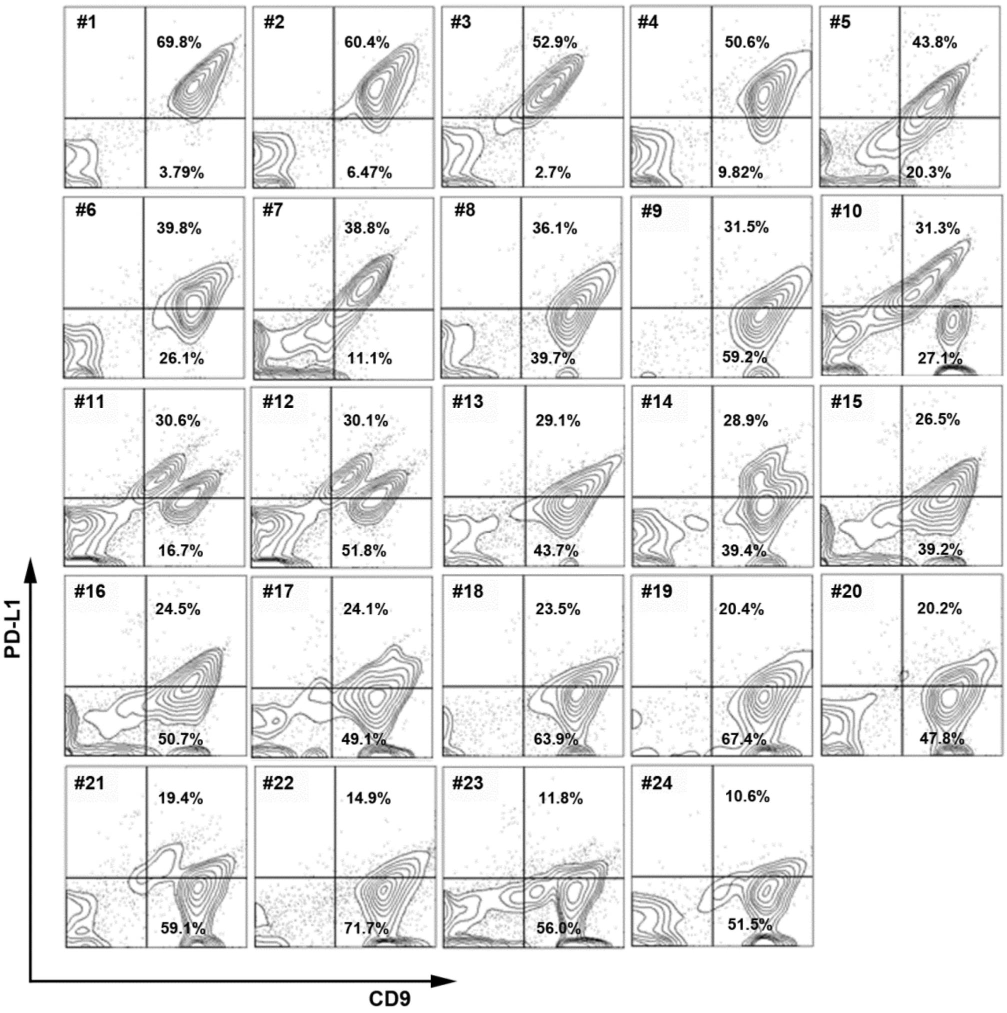

Supplementary Figure 9. PD-L1 expression of isolated exosomes from 24 patients with NSCLC. PD-L1 levels in isolated exosomes from 24 patients with NSCLC were determined using a FACScan flow cytometer. Isolated exosomes were stained with extracellular anti-human CD9 (FITC), as an exosome biomarker protein, and anti-human CD274 (PE, PD-L1). The percentage of PD-L1 positivity within the exosomes is described in the right upper quadrants. The patients were numbered according to the percentage of PD-L1-positive exosomes.

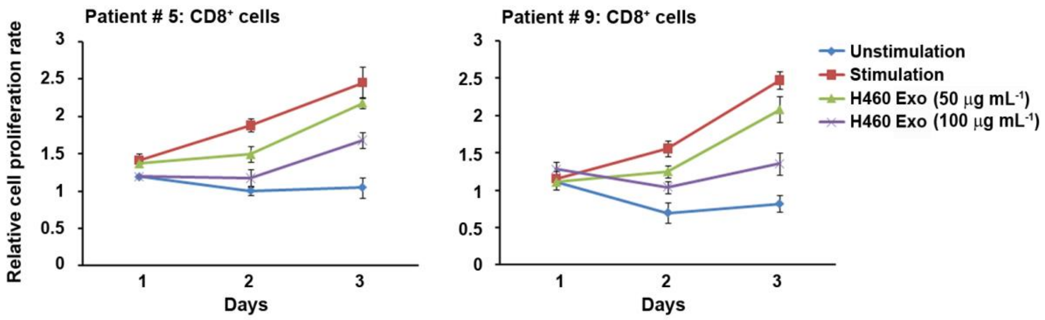

Supplementary Figure 10. Proliferation of isolated $CD8^+$ T cells from patients with NSCLC. $CD8^+$ T cells were cultured with or without H460-derived exosomes in the presence of anti-CD3, anti-CD28, and IL-2 for the indicated times. Cell proliferation was measured using a cell counting assay.
